# Resolución enfermera de los procesos leves autolimitados en atención primaria: estudio descriptivo

**DOI:** 10.1016/j.aprim.2021.102219

**Published:** 2022-05-02

**Authors:** Judit Román-Baquero, David Redondo-Collado

**Affiliations:** a Centro de Salud Casco Viejo Bilbao, OSI Bilbao-Basurto, Osakidetza, Bilbao, España; b Centro de Salud Elorrio-Berriz, OSI Barrualde-Galdakao, Osakidetza, Elorrio, España

**Keywords:** Enfermería de práctica avanzada, Evaluación de procesos, Efectividad, Atención primaria, Proceso leve autolimitado, Gestión de la demanda, Advanced practice nursing, Process assessment, Effectiveness, Primary health care, Low complexity problems, Demand management

## Abstract

**Objetivo:**

Describir la capacidad de la enfermera de Atención Primaria en la resolución de los procesos leves autolimitados (PLA), tras la implementación de la Gestión de la Demanda Asistencial (GDA) en Euskadi.

**Emplazamiento:**

Se analizaron 25 centros de salud de Atención Primaria de la OSI Bilbao-Basurto.

**Diseño:**

Estudio observacional descriptivo transversal. Utilizando como guía protocolos previamente consensuados y dentro de su ámbito competencial, la enfermera valora y resuelve cinco PLA: catarro vías altas, dolor de garganta, fiebre, náuseas y/o vómitos y diarrea. Además, puede derivar a otros profesionales los casos que detecta patología concomitante o agravantes de salud.

**Participantes:**

Se analizaron 6.985 registros de pacientes que consultaron por uno de los cinco PLA, entre el 1 de noviembre de 2019 y el 29 de febrero de 2020.

**Mediciones principales:**

La variable principal fue la resolución enfermera. Los tres modos posibles de resolución fueron: educación sanitaria, educación sanitaria y consulta administrativa médica y educación sanitaria y consulta médica.

**Resultados:**

La enfermera resolvió el 47% de los PLA. Se apreciaron diferencias en la resolución según el tipo de proceso, llegando a resolver hasta el 57% de los procesos en las consultas por diarrea. El 10,5% (IC 95%: 9,8%-11,2%) de las personas atendidas reconsultaron por motivos relacionados con el PLA de origen. Las reconsultas no guardaron relación con el modo de resolución del proceso.

**Conclusiones:**

La enfermera resuelve casi la mitad de los procesos que valora a pesar de no disponer de algunas herramientas como la competencia para indicar y dispensar medicamentos.

## Abstract (en)

**Background:**

To describe the capacity of the primary health care's nurse in the resolution of the self-limiting mild processes after the implementation of the healthcare demand's process in Basque Country.

**Setting:**

25 primary health care centers of the OSI Bilbao-Basurto.

**Type of study:**

An observational, descriptive, transversal study. Using as a guide protocols previously agreed and within hers scope of competence, the nurse values and resolves five self-limiting mild processes: upper airway infection, sore throat, fever, nausea and/or vomiting and diarrhea. Furthermore, the nurse can refer patients to other specialist in the cases concomitant disease is detected or their state of heath is aggravated.

**Participants:**

6985 patient's records, who consulted 1 of 5 self-limiting mild processes from 1st November 2019 to 29th February 2020, were analyzed.

**Main measurements:**

The main variable was the nurse's resolution. The three possible resolutions ways were health education, health education, medical administrative consultation and health education, and medical consultation.

**Results:**

The nurse solved the 47% of the self-limiting mild processes. According to the type of process, differences in the resolution were appreciated, solving as far as 57% of the processes of consultations for diarrhea. 10.5% (CI 95%; 9.8-11.2%) of the assisted people were followed-up for reasons related to the self-limiting mild processes of the origin. The follow-up consultations were not related to the way the process was resolved.

**Conclusions:**

The nurse solves nearly half of the processes that values in spite of not having some of the tools such as, the competence of indicate and dispense medications.

## Introducción

La estrategia para la atención primaria en Euskadi^1^, publicada en febrero de 2019, recoge la implantación del modelo corporativo de la Gestión de la Demanda Asistencial (GDA) en Atención Primaria (AP). Este modelo nació y se implantó en Cataluña en el año 2009^2^, extendiéndose posteriormente a otra comunidad como Andalucía. La GDA tiene como objetivo el trabajo en equipo del profesional administrativo, de enfermería y médico de AP, con el fin de dar una respuesta eficiente y proactiva a la necesidad de salud que presenta el ciudadano.

Desde el Área de Atención al Paciente o al Usuario (AAPU) se realiza un triaje administrativo con el objetivo de dirigir a cada paciente al lugar donde le puedan solucionar su necesidad. La implementación del modelo requiere que el profesional de AAPU y la enfermera desarrollen nuevas habilidades y competencias. Es la línea 4 de la estrategia, la que versa sobre las nuevas competencias asistenciales de la enfermera de AP, con el fin de convertirla en un referente en el ámbito asistencial en colaboración con el médico.

Tradicionalmente, la cartera de servicios del profesional de enfermería en el Servicio Vasco de Salud incluía la atención a las personas (y sus cuidadores) con patologías crónicas, la promoción de la salud y prevención de enfermedades y la asistencia a accidentes y daños como heridas, picaduras, etc. En el contexto actual, entre otras, la enfermera adquiere la competencia de valorar cinco procesos leves autolimitados (PLA) catarro de vías altas, fiebre, dolor de garganta, náuseas y/o vómitos y diarrea. Se considera PLA^3^, ^4^, ^5^, ^6^, ^7^ al conjunto de signos y síntomas que refiere el paciente y que tienden a resolverse espontáneamente. La enfermera, utilizando como guía protocolos previamente consensuados y dentro de su ámbito competencial, resuelve estos procesos agudos centrándose en la educación sanitaria, el autocuidado y la desmedicalización. En aquellos casos en los que detecte patología concomitante o agravantes de salud puede derivar el caso a otros profesionales.

Existen pocos estudios sobre el tema^8^, ^9^, ^10^ y los resultados muestran mucha variabilidad en cuanto a resolución enfermera. Los problemas que recogen los estudios difieren en número y en tipo de procesos, reportan mejores resultados en resolución autónoma aquellos que incluyen problemas habitualmente tratados por las enfermeras como heridas o quemaduras y los que la enfermera recibe formación en la resolución de procesos^10^. A pesar de la variabilidad, todos coinciden en que la GDA fomenta la educación sanitaria, aumenta el tiempo que el profesional dedica al paciente, desmedicaliza problemas y supone una oportunidad de desarrollo para la profesión enfermera^8^, ^9^, ^11^. El Servicio Vasco de Salud implementó completamente la GDA a mediados del año 2019. Después, no existen estudios en Euskadi que describan cual es el impacto en resolución que puede tener el desarrollo de esta nueva competencia enfermera en las consultas de AP.

El objetivo principal del estudio fue describir la capacidad de la enfermera de AP en la resolución de los PLA. Los objetivos secundarios fueron evaluar si existía diferencia entre el tipo de PLA y el modo de resolución, analizar el motivo por el que la enfermera precisó ayuda de otro profesional y examinar si el paciente reconsultó por el mismo motivo en las 72 horas siguientes en AP, Punto de Atención Continuada (PAC) o Servicio de Urgencia del Hospital (SUH).

## Material y métodos

### Diseño del estudio

Estudio descriptivo trasversal.

### Contexto

Los datos fueron recogidos entre el 1 de noviembre de 2019 y el 29 de febrero de 2020 en los 25 centros de salud de AP que componen la OSI Bilbao-Basurto y que dan cobertura sanitaria a 347.298 habitantes. Este estudio obtuvo el informe favorable del Comité Ético de Investigación del Hospital de Basurto.

### Población de estudio

Los criterios de inclusión fueron: edad mayor de 14 años, demanda de valoración sanitaria por uno de los cinco PLA de adultos: catarro de vías altas, dolor de garganta, náuseas y/o vómitos, diarrea y fiebre. Estar citados en agenda de enfermería de adultos y tener cumplimentado de forma correcta el registro en la historia clínica informatizada del Servicio Vasco de Salud Osakidetza (Osabide Global AP). Se excluyeron a los pacientes mayores de 14 años que fueron valorados por un PLA en pediatría, a los que solicitaron asistencia por PLA en un PAC, así como a los que tenían el formulario de registro incompleto.

### Variables

La variable dependiente principal fue la resolución enfermera de los PLA. Con base en los protocolos de los PLA^3^, ^4^, ^5^, ^6^, ^7^, los tres modos de resolución fueron:

*Educación sanitaria:* La valoración e intervención enfermera es suficiente para resolver el problema de salud que presenta el paciente.

*Educación sanitaria y consulta administrativa médica:* La valoración e intervención enfermera precisa, además, una actuación de gestión médica, basada, generalmente, en prescripción de fármacos que componen el botiquín casero y en la expedición de incapacidades temporales.

*Educación sanitaria y consulta médica:* La enfermera tras la valoración del problema, detecta una serie de criterios o agravantes de salud que hacen necesaria una derivación médica para valoración y/o asistencia sanitaria más compleja.

Se consideró PLA resuelto por la enfermera los dos primeros modos de resolución. El modo de resolución de educación sanitaria y consulta administrativa médica se introdujo porque, en su mayoría, responde a trámites burocráticos que la enfermera no está autorizada a realizar y a que la actuación de gestión médica esta tramitada por el profesional de enfermería. La enfermera no resolvió el PLA cuando derivó el paciente al MAP para valoración.

La variable dependiente secundaria fue la reconsulta en AP, PAC o SUH por el mismo motivo que el PLA de origen dentro de las 72 horas siguientes a la primera consulta.

Las variables independientes fueron:

• Tipo de PLA
• Edad
• Sexo del paciente
• Criterio de derivación médica: se establecieron seis categorías que recogían los motivos de no resolución autónoma (tabla 1).

**Tabla 1:** Motivos de derivación al médico indicado por los protocolos según Proceso Leve Autolimitado (PLA)

Criterio derivación	Catarro	Dolor de garganta	Fiebre	Nauseas/vómitos	Diarrea
ANTECEDENTES PERSONALES (AP)	Enf. respiratorias (asma EPOC) Enf. Cardiacas Insuficiencia renal Inmunosupresión Asplenia Pérdida de peso involuntaria Embarazo	Inmunosupresión/ Asplenia Enf. Cardiacas Insuficiencia renal Enf. respiratorias (asma, EPOC) Embarazo	Enf. respiratorias (asma EPOC) Enf. Cardiacas Diabetes Hepatopatía Insuficiencia renal Inmunosupresión Asplenia Obesidad mórbida (IMC > 40) Embarazo o 2 semanas postparto < 18 años y tratamiento con AAS Viaje en los 6 meses previos a un destino tropical Picadura de garrapata Ingreso hospitalario y/o IQ en 7 días previos Tto. activo con radio/quimioterapia	Bulimia u otro trastorno de la alimentación Vértigo Diabetes tipo I Tto. con anticoagulantes orales Tto. activo con radioterapia/quimioterapia Embarazo Tto. con anticonceptivos orales Otras personas del entorno afectadas Viaje en los 6 meses previos a un destino tropical. Sospecha de intoxicación alimentaria	Enf. cardiaca Inmunosupresión/asplenia Diabetes tipo I Enfermedades autoinmunes Insuficiencia renal Tto. con anticonceptivos orales Embarazo Tto. con anticonceptivos orales Otras personas del entorno afectadas Viaje en los 6 meses previos a un destino tropical. Sospecha de intoxicación alimentaria Tto. activo con radio/quimioterapia
PROCESO LEVE AUTOLIMITADO (PLA)	> 10 días de evolución Disnea Otalgia intensa Sospecha de rinosinusitis (cefalea frontal, rinorrea, hiposmia) Expectoración hemoptoica, purulenta y/o fétida Tos persistente Gran afectación del estado general Fiebre > 38°C > 72 h Dolor costal	Alteración importante de la deglución Signos y síntomas de obstrucción respiratoria sospecha de infección o absceso en espacio retrofaríngeo (tortícolis, rigidez nucal, trismus, dolor de garganta severo unilateral) Erupciones o manchas en la piel Afectación importante del estado general > 7 días de evolución	Disnea Sospecha de afectación cardiaca (mareo, sudor frio, palidez de extremidades…) Sospecha de afectación del sistema nervioso central (confusión, desorientación, convulsión, somnolencia, rigidez de nuca…) Dolor abdominal y fiebre/febrícula (tª > 37°C) Erupciones o manchas en la piel Fiebre > 38°C > 72 h Dolor costal Diarrea	Signos de deshidratación Intolerancia oral absoluta Dolor abdominal intenso Vómitos frecuentes (> 5/12 h) Vómitos biliosos y/o sangre Vómitos en escopetazo Sospecha de obstrucción Vómitos en escopetazo Dolor abdominal y fiebre/febrícula > 24 h Evolución > 6 días	Gran afectación del estado general Signos de deshidratación Fiebre > 38°C Dolor abdominal severo Deposiciones con moco, pus o sangre > 7 días a pesar de medidas conservadoras > 5 deposiciones en 24 h
NO PLA	Motivo diferente a los 5 PLA	Motivo diferente a los 5 PLA	Motivo diferente a los 5 PLA	Motivo diferente a los 5 PLA	Motivo diferente a los 5 PLA
A CRITERIO ENFERMERA	La enfermera decide derivación médica aunque no cumple criterios de derivación.	La enfermera decide derivación médica aunque no cumple criterios de derivación.	La enfermera decide derivación médica aunque no cumple criterios de derivación.	La enfermera decide derivación médica aunque no cumple criterios de derivación.	La enfermera decide derivación médica aunque no cumple criterios de derivación.
NO GESTIONABLE	El paciente demanda valoración por médico.	El paciente demanda valoración por médico.	El paciente demanda valoración por médico.	El paciente demanda valoración por médico.	El paciente demanda valoración por médico.
ADMINISTRATIVA	Gestión IT o prescripción	Gestión IT o prescripción	Gestión IT o prescripción	Gestión IT o prescripción	Gestión IT o prescripción

Enf.: enfermedad; Tto.: tratamiento; IT.: incapacidad temporal.

## Fuentes de datos

Los datos sociodemográficos se obtuvieron a través de la Unidad de Información Estadística de la OSI Bilbao-Basurto mediante la plataforma OBI y se trasladaron de forma seudonimizada a los investigadores en formato Excel. Aquellos datos que no pudieron ser extraídos de OBI, como fue el análisis de la nueva cita en AP o PAC en las 72 horas siguientes y su relación con el PLA de origen, la Unidad de Investigación y Docencia en Enfermería de la OSI Bilbao-Basurto se encargó de revisar las historias, recoger los datos y anonimizarlos. Para el análisis de las reconsultas en el SUH, se obtuvo el CIE 10 y se descartaron las reconsultas derivadas desde AP o PAC.

### Tamaño muestral y procedimiento de muestreo

De las 8.354 personas que solicitaron valoración por 1 de los cinco PLA y tras aplicar los criterios de inclusión y exclusión, se obtuvo una población de estudio de 6.985 personas. El análisis de los datos (n = 6.985) mostró que 1.077 personas tenían nueva cita para consulta en AP o PAC en las 72 horas siguientes a la consulta por PLA. Para conocer si existía relación entre esta citación y el PLA de origen, se precisó realizar una revisión de historias clínicas. Debido a la imposibilidad de consultar tal número de historias se realizó un muestreo aleatorizado simple.

Dada la escasa evidencia previa (situada en torno al 11%^8^, ^11^) se presupuso una proporción del 32%. Para dicha proporción, un nivel de confianza del 95% y una precisión del 5%, se necesitaron 284 personas, que fueron seleccionados mediante una secuencia de números aleatorios obtenida del programa informático Epidat 4.2.

Finalmente, se recogieron los datos de 283 historias clínicas, quedando uno fuera de estudio por error de consulta de historia.

Las medidas cuantitativas fueron descritas mediante media y desviación estándar (DE). Las variables cualitativas se resumieron mediante porcentajes, utilizando el test *X*^*2*^ para la comparación de proporciones. Se consideró un resultado estadísticamente significativo cuando p < 0,05. El análisis de datos se llevó a cabo con el paquete estadístico PSPP 0.8.5 y la hoja de cálculo Excel 2013.

## Resultados

**Figura 1:**
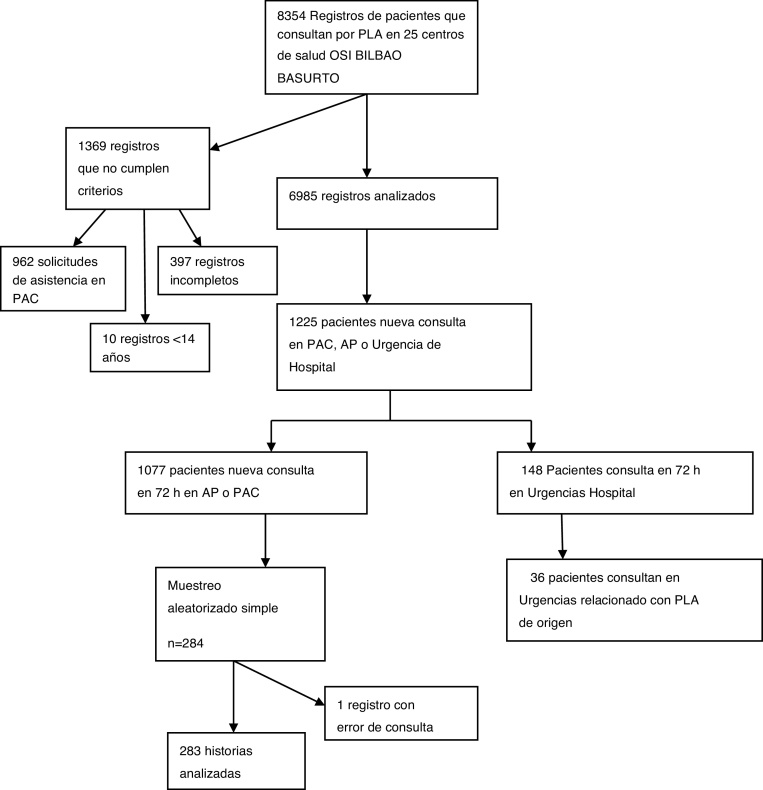
Esquema general del estudio.

De las 8.354 personas que solicitaron valoración por uno de los cinco procesos, entre el 1 noviembre de 2019 y el 29 de febrero de 2020 se seleccionaron 6.985 registros de pacientes; 962 registros (11,5%) fueron eliminados porque correspondían a personas que habían demandado valoración por PLA en un PAC, 397 registros (4,7%) estaban incompletos y 10 (0,1%) eran de pacientes menores de 14 años (fig. 1). La edad media de las personas atendidas fue de 46,6 (DE: 20) años siendo el 57,4% mujeres.

**Figura 2:**
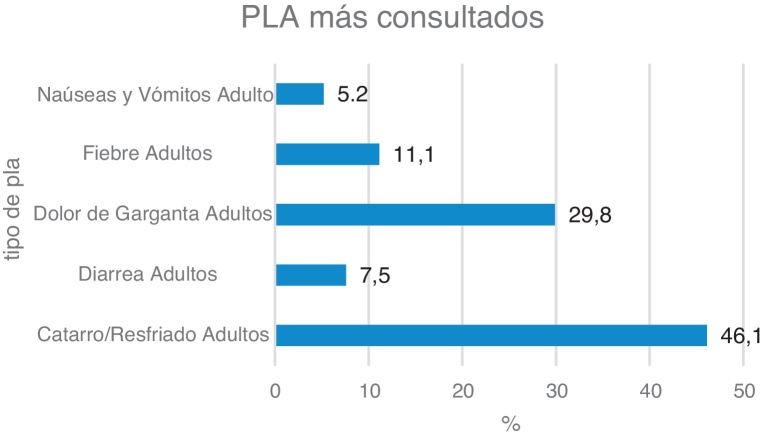
Porcentaje de consulta según tipo de PLA. n = 6.985.

**Figura 3:**
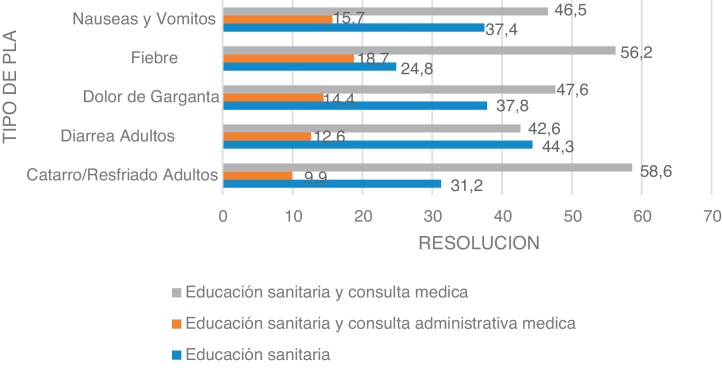
Porcentajes de resolución según tipo de PLA. n = 6.985.

El PLA por el que más consultas se realizaron fue el de catarro de vías altas (46,1%), seguido del dolor de garganta (29,9%) (fig. 2). Las enfermeras resolvieron el 47% de los PLA, existiendo diferencias estadísticamente significativas entre la capacidad resolutiva de la enfermera y el tipo de PLA realizado (p < 0,001). Los profesionales de enfermería solucionaron de forma autónoma el 57% de los casos en el proceso de diarrea, el 53% de náuseas y vómitos y el 52% de las consultas por dolor de garganta (fig. 3).

**Figura 4:**
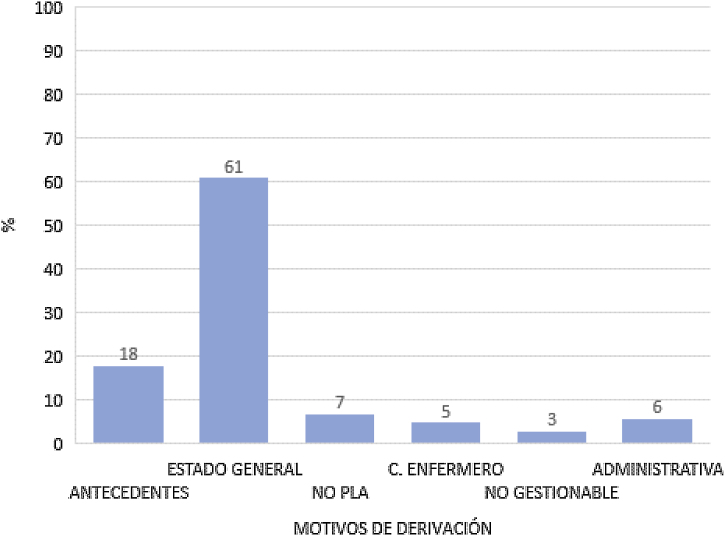
Porcentajes según motivos de derivación al médico de los PLA no resueltos por la enfermera. n = 2.928.

Se registraron los motivos de derivación al médico en 2.928 registros (79%) de PLA no resueltos por la enfermera. El motivo más frecuente respondió a la sintomatología y estado general derivado del propio proceso que presentaba el paciente (61%). La comorbilidad supuso el 18% de las derivaciones, el 6% se debió a temas administrativos (gestión de incapacidades temporales y prescripción de fármacos), un 5% a criterio de la enfermera y un 3% se negó a ser valorado por la enfermera (fig. 4).

El 10,5% (IC 95%: 9,8-11,2%) (962 personas) de las personas atendidas reconsultaron por motivos relacionados con el PLA de origen en AP o PAC en las 72 h siguientes. De las 283 historias de personas analizadas que reconsultaron en AP o PAC, 192 estaban relacionadas con el PLA de origen. El 45,5% fueron del grupo de resolución Educación sanitaria y el 38% del de Educación sanitaria y consulta médica. No se encontró significación estadística entre la reconsulta y el modo de resolución (p = 0,454). Del 10,5% de las reconsultas, el 6,7% (IC 95%: 6,1-7,3%) lo fueron por motivos clínicos y el 3,8% (IC 95%: 3,3-4,2%) consultas administrativas. 148 pacientes (3,9%) reconsultaron en SUH. De estos, 36 (0,5%) por motivos relacionados con el PLA de origen.

## Discusión

La implementación de la GDA ha acarreado el desarrollo de nuevas competencias enfermeras, entre las que se encuentra la valoración de los PLA. El profesional de enfermería resuelve de forma autónoma el 47% de los PLA atendidos. Existen diferencias importantes en cuanto a la resolución de PLA en la bibliografía existente dado que, los porcentajes de resolución oscilan entre el 15,3 y el 86,3%^8^, ^9^, ^10^, ^11^. Esta variabilidad en los resultados puede deberse a diferentes motivos tales como, la formación y entrenamiento en la resolución de problemas agudos^8^, ^9^, ^10^, ^12^, la indicación, uso y dispensación de medicamentos de forma autónoma por la enfermera^13^ (en desarrollo en Euskadi en el momento de estudio) y la experiencia en la resolución de estos procesos agudos leves.^8^, ^10^ Además, aquellos estudios que incluyen procesos como heridas y quemaduras registran mejores resultados de resolución autónoma enfermera^8^, ^9^.

De las 8.354 personas que demandaron asistencia durante el periodo de estudio, se descartaron 397 registros (4,7%) porque el formulario estaba incompleto y 962 registros por solicitar asistencia en un PAC. Resulta llamativo que el 11,5% de las personas solicitaran valoración por un proceso leve en un PAC de forma urgente y no en su centro de salud con cita previa, sería interesante conocer si estas consultas responden a causas verdaderamente indemorables.^14^

Los resultados del estudio muestran variabilidad en cuanto a la resolución enfermera, según el tipo de PLA. La enfermera resuelve más frecuentemente las consultas realizadas por el PLA diarrea, náuseas y vómitos y dolor de garganta. Catarro de vías altas es el PLA más consultado (46%), siendo a su vez el que menos resolución autónoma tiene. Estos resultados coinciden con otros estudios^9^, ^11^, ^12^, ^15^ y pueden deberse, a que la recogida de datos se realiza coincidiendo con una época de alta incidencia de catarro de vías altas y que su resolución, puede requerir una valoración física más compleja de la que realiza la enfermera habitualmente^9^.

Sólo el 6,7% de las reconsultas fueron por motivos clínicos, el resto se debieron a aspectos administrativos (prescripciones, tramites de alta o baja laboral). Estos datos muestran cifras más bajas de reconsulta que en estudios similares^8^. Lo mismo ocurre en el caso de las reconsultas en el Servicio de Urgencias del Hospital. A diferencia de otros estudios, se constató que no existe relación entre el modo de resolución que la enfermera da al PLA que atiende y la reconsulta.

Resulta llamativo que el 6% de los PLA no resueltos se debiera a aspectos administrativos, es decir, trámites como incapacidades temporales y prescripción de fármacos del botiquín casero y que dispone de un modo de resolución propio para ello. El 5% fue derivado a criterio de la enfermera. En estos casos la enfermera no resolvió el proceso, aunque el motivo de derivación recogido no justificaba la derivación. Una posible explicación puede ser la falta de experiencia y empoderamiento en la resolución de los PLA ya que, la recogida de datos comienza cuatro meses después de la implementación total y los profesionales de enfermería recibieron escasa formación antes de enfrentarse al desarrollo de esta nueva competencia. Un 3% de los pacientes se negaron a que el proceso que presentaban fuera valorado por una enfermera. Cabe destacar que es un porcentaje extremadamente bajo, pero se puede hallar explicación en que estos procesos han sido tradicionalmente valorados por los profesionales médicos y que las campañas para sensibilizar e informar a la población sobre esta nueva competencia de la enfermera fueron escasas. Mayoritariamente, los pacientes han sido informados del cambio por los profesionales de las AAPU de su centro de salud al solicitar la cita.

La falta de empoderamiento de la enfermera en la resolución de los PLA y la reticencia de algunos profesionales por ver la GDA como una invasión de competencias han sido algunas de las dificultades a los que ha tenido que hacer frente la implementación del modelo GDA en el inicio^16^. Resultaría interesante realizar un nuevo estudio cuando además de la experiencia adquirida por la práctica diaria, el profesional de enfermería pueda indicar, usar y dispensar medicamentos dentro de su práctica habitual.

Existen varias limitaciones en el estudio, por un lado, la escasez de estudios previos sobre el tema, por otro, sólo se recogió el motivo de derivación del 79% de los PLA no resueltos. Además, se consideró la resolución de cinco procesos leves que tradicionalmente y hasta el momento de implantación, habían sido valorados por el profesional médico. No se tuvieron en cuenta problemas como heridas, quemaduras o tapones óticos, muy comunes en la práctica diaria enfermera y con alta resolución autónoma, llegando en algunos casos hasta el 94-100% de resolución^9^. Por último, la información obtenida a través de la Unidad de Información Estadística sólo recogió los datos de los pacientes que reconsultaron en el SUH de Basurto (Bilbao). No fue posible conocer si solicitaron nueva consulta en otro Servicio de Urgencia Hospitalaria de la red pública o privada.

En conclusión, la enfermera cierra de forma autónoma casi la mitad de los PLA que atiende, a pesar de llevar poco tiempo desarrollando la competencia y no disponer de la herramienta de indicación, uso y dispensación enfermera. Las reconsultas relacionadas con el PLA de origen en AP, PAC o SUH en las 72 horas siguiente, no guardan relación con el modo en que la enfermera resolvió el proceso.

La GDA supone una oportunidad de desarrollo del potencial de la enfermera, permite responder a las necesidades de las personas de forma más eficiente y dar unos cuidados de mayor calidad, centrados en la desmedicalización y el autocuidado.

**Lo conocido sobre el tema**

• La Gestión de la Demanda fomenta la educación sanitaria, aumenta el tiempo que el profesional dedica al paciente y desmedicaliza problemas leves de salud.
• La puesta en marcha de la Gestión de la Demanda supone un desarrollo en el ámbito competencial de la enfermera.
• Los estudios existentes sobre el tema son escasos y los resultados muestran una gran variabilidad.

**Qué aporta este estudio**

• La enfermera resuelve casi la mitad de los Procesos Leves Autolimitados que valora, aumentando este porcentaje cuando se trata de los procesos: diarrea, náuseas y/o vómitos y dolor de garganta.
• Existe una baja prevalencia de reconsulta por un motivo relacionado con el Proceso Leve Autolimitado de origen en las siguientes 72 horas
• No existe relación entre el modo de resolución del Proceso Leve Autolimitado y la reconsulta.

## Financiación

Este trabajo ha recibido financiación de la OSI Bilbao-Basurto (OSI BB20/008) y su publicación ha sido financiada por la Comisión de Investigación de la OSI Bilbao-Basurto.

## Conflicto de intereses

Los autores declaran no tener ningún conflicto de intereses.
